# Supplementary material for: Microbiome characterization by high-throughput transfer RNA sequencing and modification analysis
Source: Nat Commun. 2018 Dec 17;9:5353. doi: 10.1038/s41467-018-07675-z (PMC6297222; doi:10.1038/s41467-018-07675-z)
Supplement: Supplementary file 1 — Supplementary Information [file 41467_2018_7675_MOESM1_ESM.pdf]

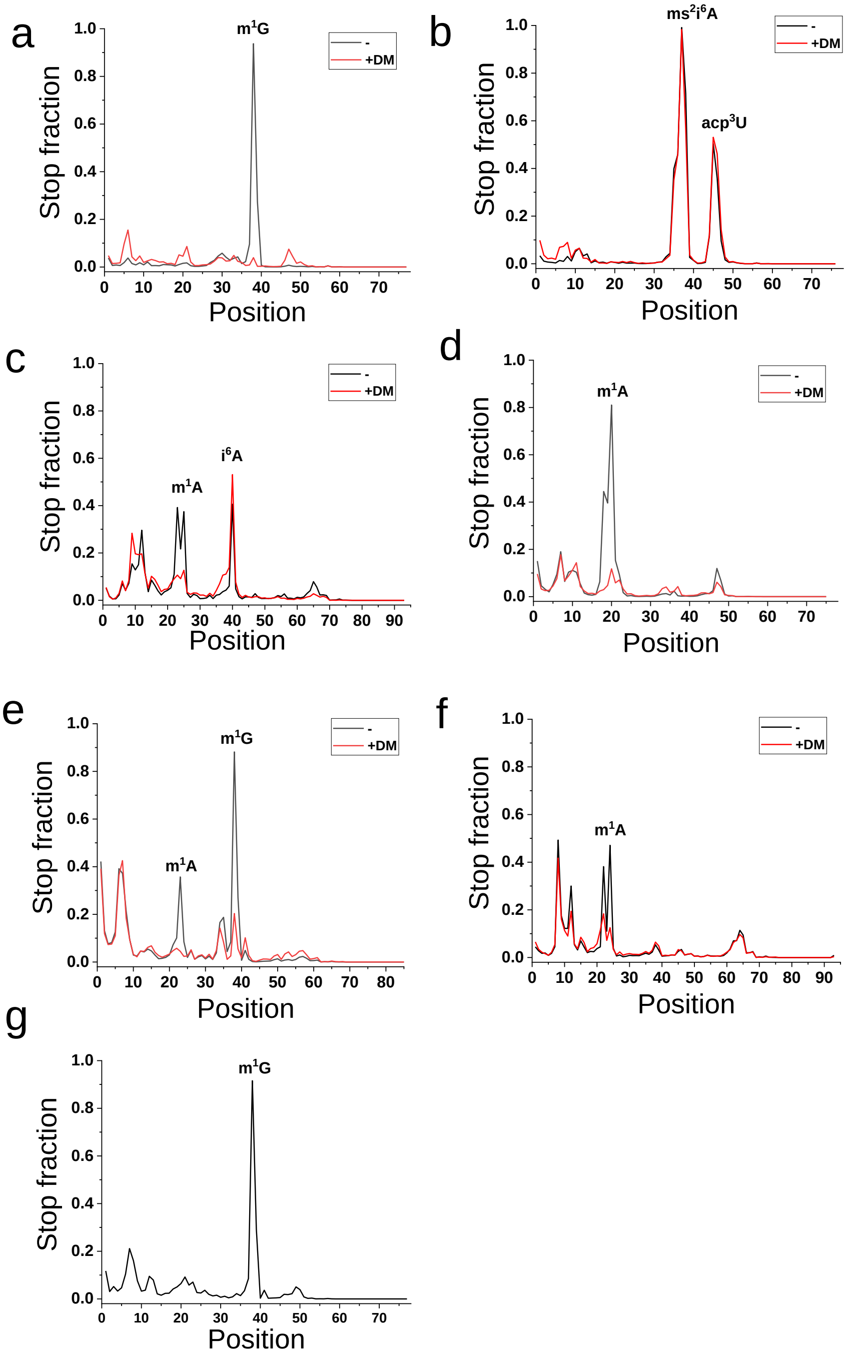

Supplementary Fig. 1: tRNA modifications of bacterial cultures from RT stops. Stop fractions are shown for the same tRNAs in Figure 1. Seq-data without demethylase treatment is in black, with demethylase treatment (+DM) in red. Called modifications are indicated by circles. (a) *E. coli* tRNA^{Pro}(CGG), m^{1}G37. (b) *E. coli* tRNA^{Phe}(GAA), acp^{3}U47, ms^{2}i^{6}A37. (c) *B. subtilis* tRNA^{Ser}(UGA), i^{6}A37, m^{1}A22. (d) *B. subtilis* tRNA^{Glu}(UUC), m^{1}A22. (e) *S. aureus* tRNA^{Leu}(UAG), m^{1}G37, m^{1}A22. (f) *S. aureus* tRNA^{Ser}(GCU), m^{1}A22. (g) *B. viscericola* tRNA^{Arg}(ICG), m^{1}G37.

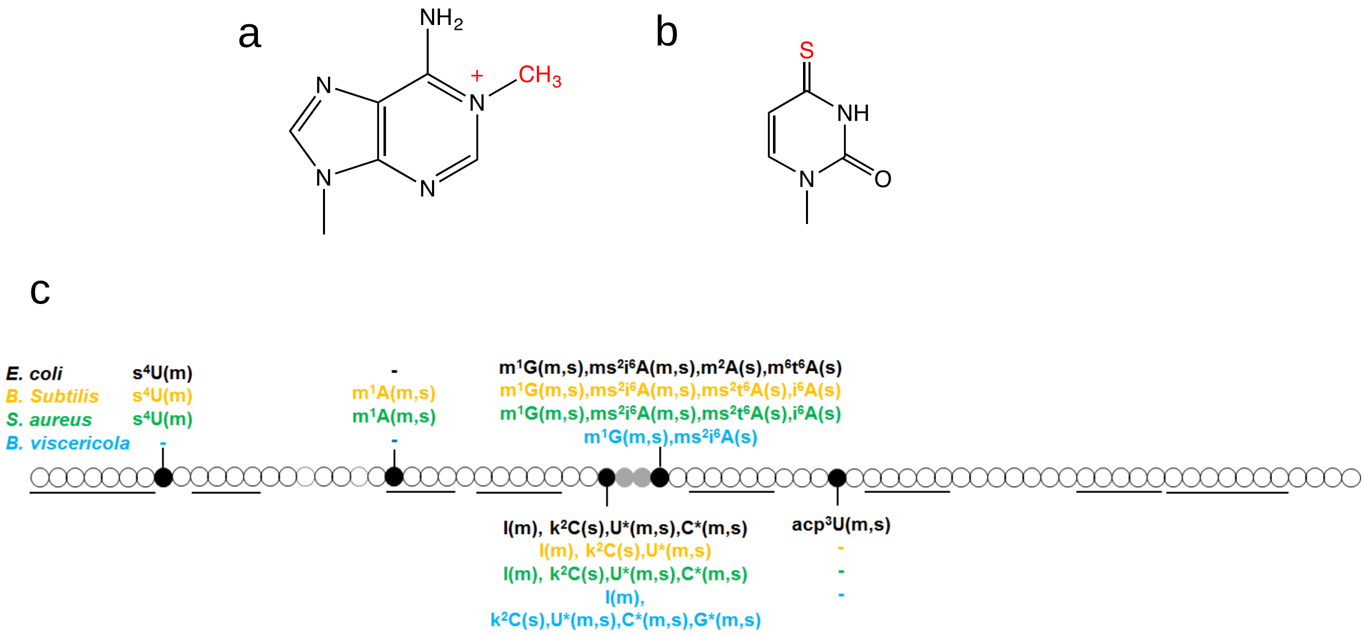

Supplementary Fig. 2: Overall pattern of modifications detected by DM-tRNA-seq in bacterial cultures. (a) chemical structure of N1-methyladenosine (m^1A). (b) Chemical structure of 4-thiouridine (s^4U). (c) Base paired regions in the tRNA secondary structure are underlined. Anticodon nucleotides are in gray circles. The four bacteria are color coded. tRNA positions with known Watson-Crick face modifications in *E. coli* and *B. subtilis* are in filled circles; they include s^4U8, m^1A22, a large number of 34 (wobble position) and 37 modifications, and acp^3U. (m) and (s) represents modification identification using mutation or stop, respectively in DM-tRNA-seq. Modification types of *S. aureus* and *B. viscericola* are inferred from the known *E. coli* and *B. subtilis* modifications.

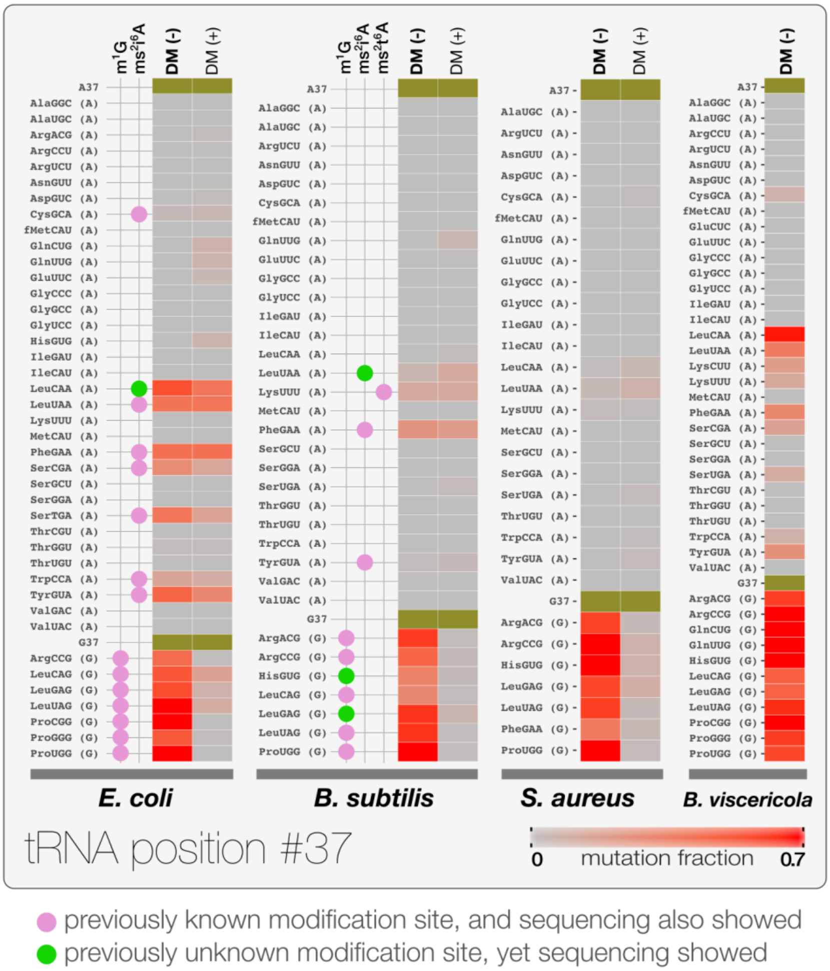

Supplementary Fig. 3: Heatmap of mutation fractions of tRNA position 37. tRNAs with different anticodons are grouped by their sequences at the respective position of modification (in parenthesis) and in alphabetical orders. Only *E. coli* and *B. subtilis* tRNA modifications have been mapped previously by 2D-TLC and LC/MS, but the mapping was not done for every tRNA species. *E. coli* and *B. subtilis* tRNA species with mutation fraction at 10-times above background is marked with a circle on the left with the following designations: Purples correspond to those known to be present and also identified by sequencing here; greens correspond to those not mapped previously but identified by sequencing.

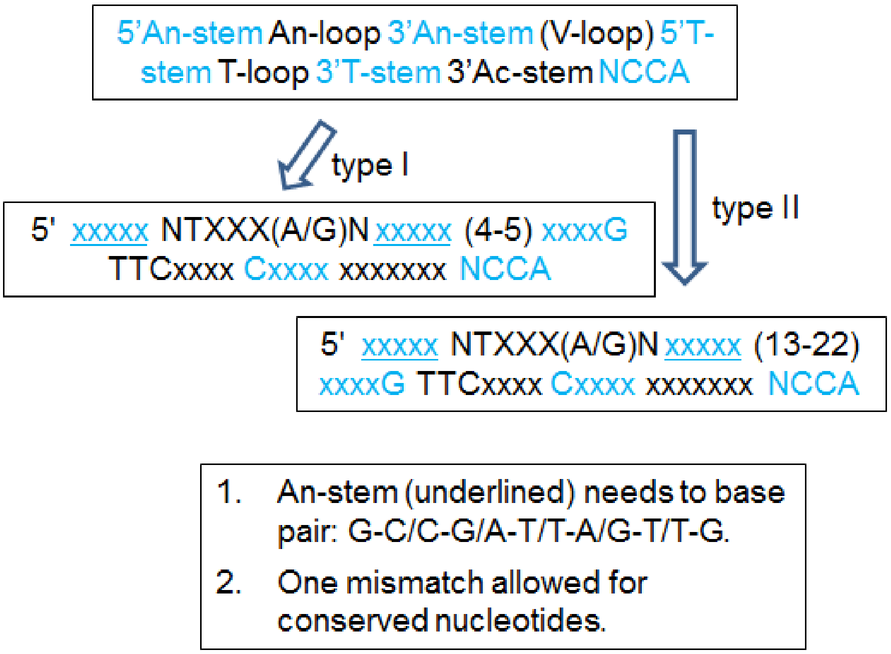

Supplementary Fig. 4: Definitions used in *de novo* identification of tRNAs in the sequencing data. Type I and type II tRNAs differ by having a short (I) or long (II) variable loop. The locations of the conserved nucleotides in the tRNA secondary structure are shown in Fig. 3a. Additional criteria for the search include the base pairing rule of the anticodon stem and allowing one mismatch among the conserved nucleotides.

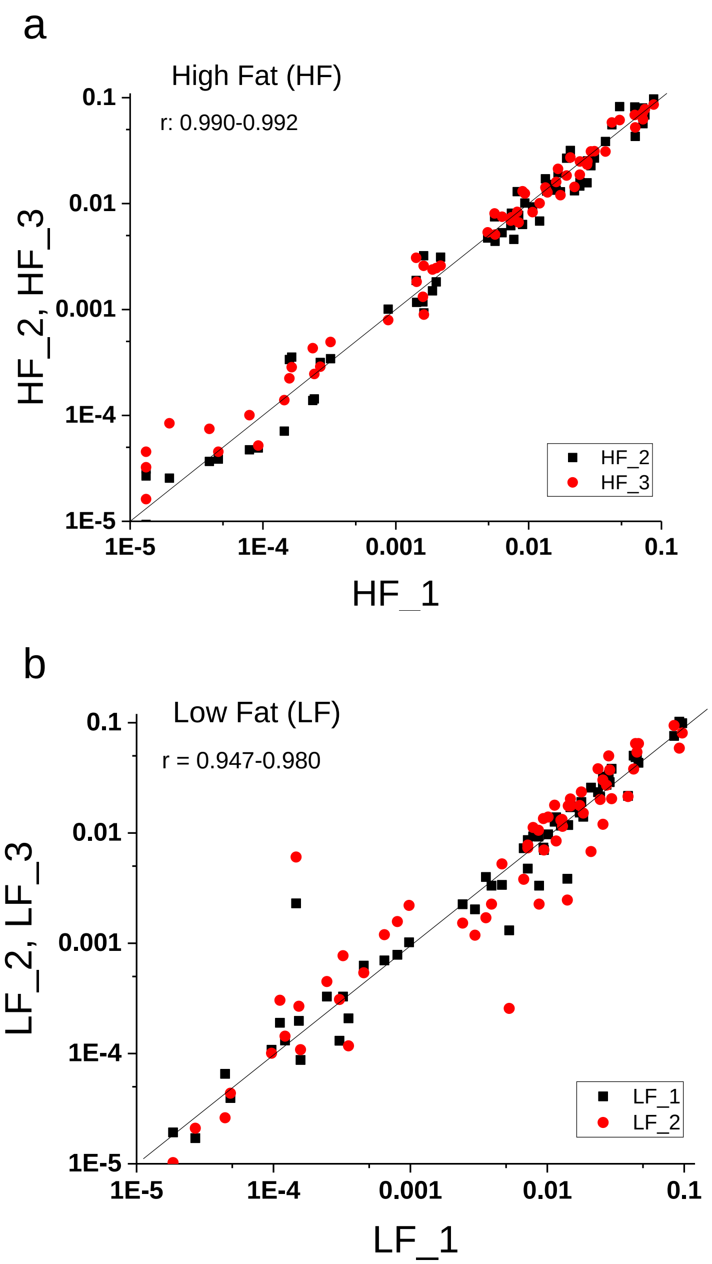

Supplementary Fig. 5: HF or LF sample relationship plots of tRNA abundance grouped by anticodons. Demethylase treated samples are shown. Line shows the diagonal of the plot. The very low abundant anticodon groups generally have A in the wobble position which is rare in the bacterial tRNA genes. These very low abundant anticodon groups may in part be derived from mutations induced by modifications at the wobble position or sequencing errors.

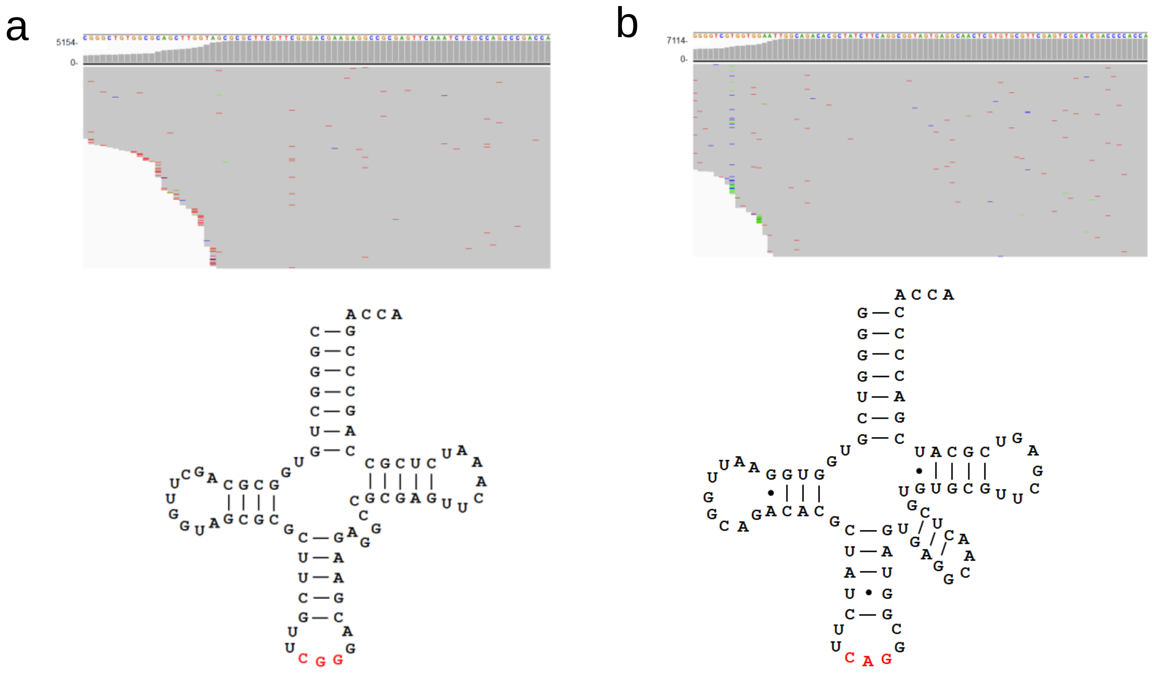

Supplementary Fig. 6: Examples of aligning tRNA sequencing reads. A type I tRNA^{Pro}(CGG) sequence (a) and a type II tRNA^{Leu}(CAG) seed sequence (b) obtained from *de novo* tRNA sequence analysis. One mismatch was allowed in the alignment. The tRNA^{Pro} is assigned to *Bifidobacterium* (class Actinobacteria). The tRNA^{Leu} is assigned to *Faecalibaculum* (class Erysipelotrichia).

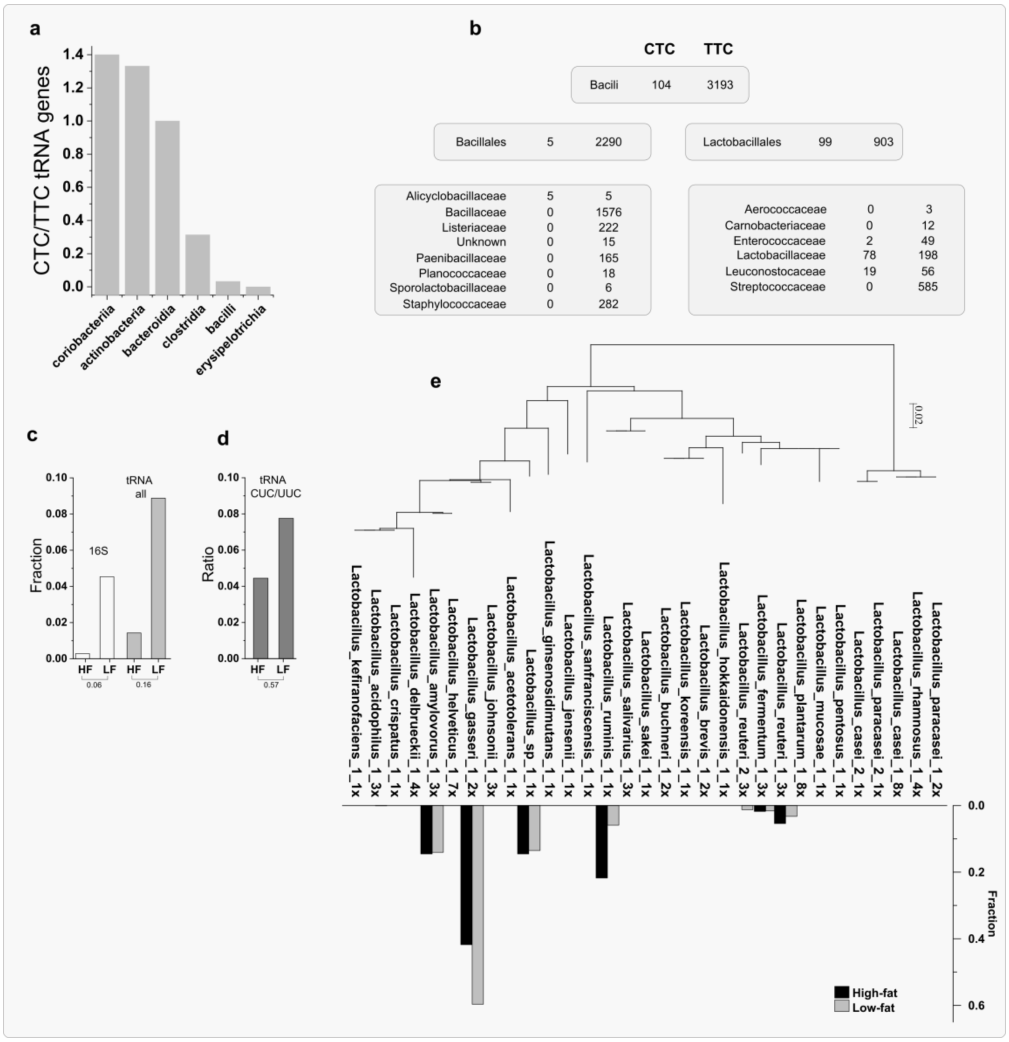

Supplementary Fig. 7: Additional analysis of anticodon-based tRNA expression taxonomy. Results are for tRNA^{Glu}(CUC) and tRNA^{Glu}(UUC). (a) The ratio of CTC/TTC genes in the tRNA gene database used in our tRNA taxonomy analysis for the six major classes present in the mouse cecum microbiome. (b) Further division of CTC/TTC genes in Bacilli at the level of class, order and family. CTC genes are concentrated in one single family of Lactobacillaceae. (c) Lactobacillaceae taxonomy based on 16S-seq (open bars), tRNA-seq from all anticodons (light gray bars). (d) Relative expression of tRNA^{Glu}(CUC) over tRNA^{Glu}(UUC) of Lactobacillaceae by tRNA-seq. (e) Lactobacillus is the only genus found in the Lactobacillaceae family by tRNA-seq in mouse cecum. Among the 31 CTC gene groups in Lactobacillus, some are present in multiple copies (marked by _nx). ClustalX alignment shows 18 different sequences among the 31 groups. Six of 18 sequences can be identified in the tRNA-seq data; the proportion of each is shown in dark (HF) and gray (LF) bars.

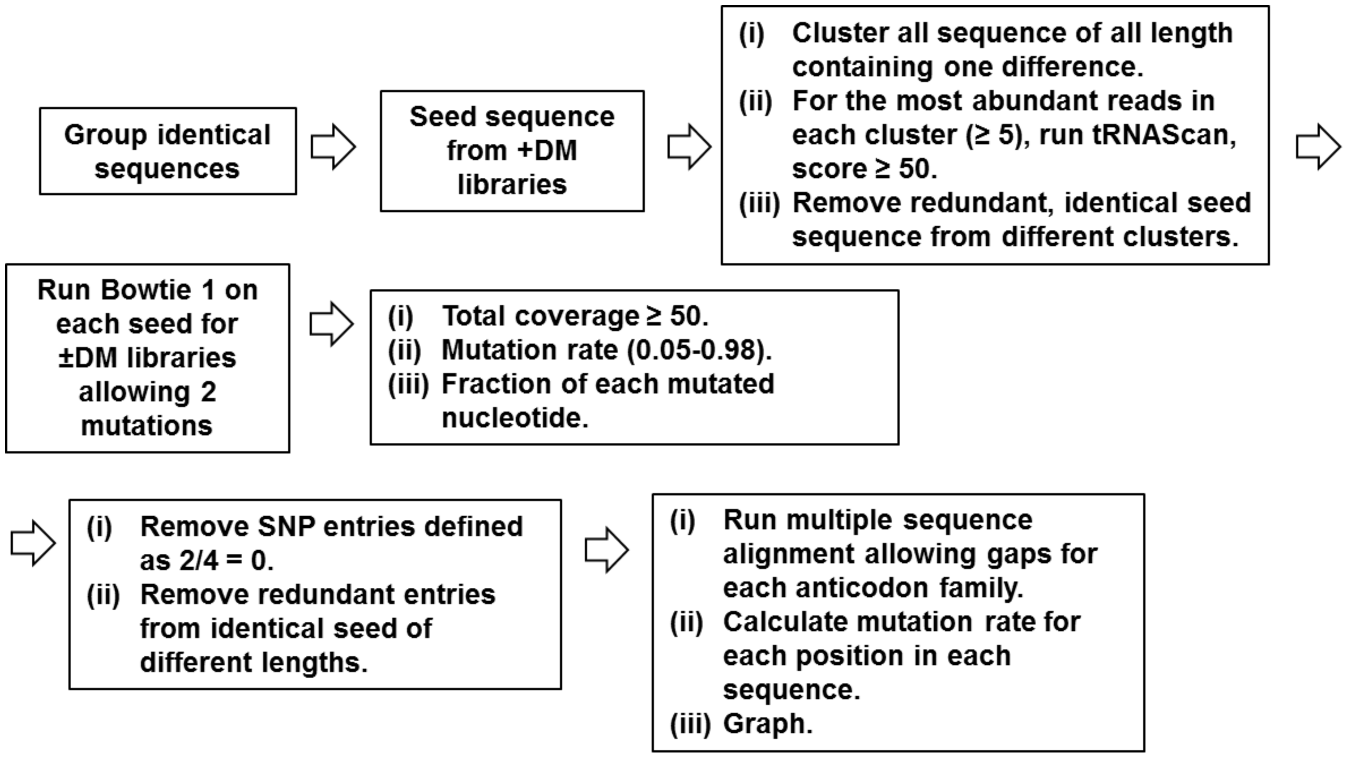

Supplementary Fig. 8: Step-by-step flow diagram for the analysis of microbiome tRNA modifications.

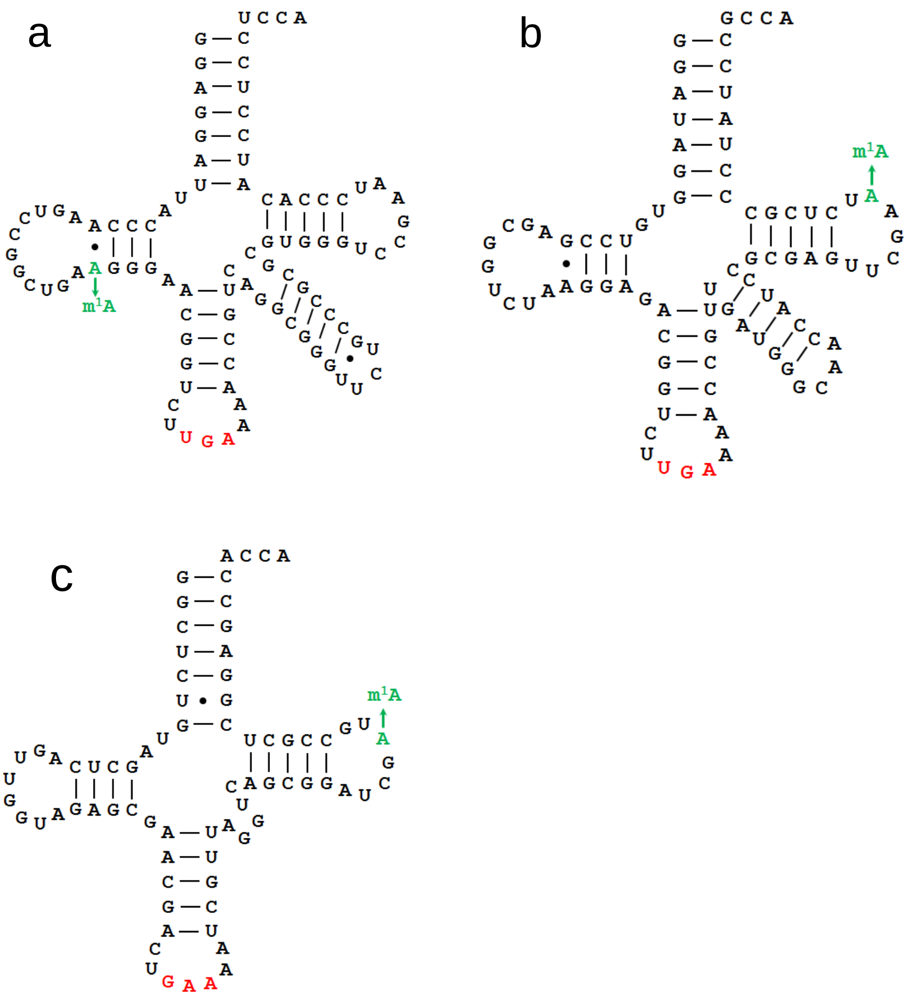

Supplementary Fig. 9: Examples of tRNA sequences containing m^{1}A modification. (a) m^{1}A22, tRNA^{Ser}(UGA), *Lactobacillus*; (b) m^{1}A59, tRNA^{Ser}(UGA), *Bifidobacterium*; (c) m^{1}A58, tRNA^{Phe}(GAA), *Bifidobacterium*.

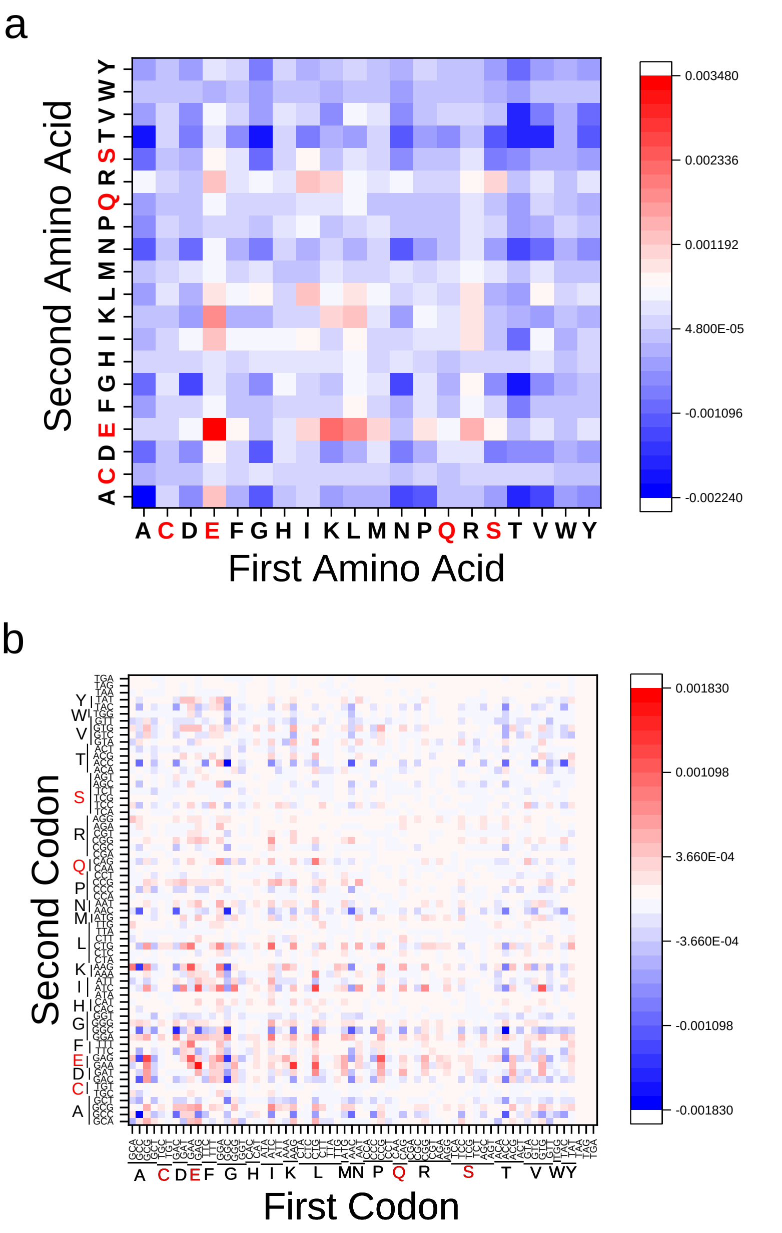

Supplementary Fig. 10: Amino acid and codon pair analysis of the day 29 meta-proteomics data. Same as Figure 7f (panel a here) and 7g (panel b here), except using day 29 data.

Supplementary Table 1: Anticodon loop modifications in bacterial cultures by DM-tRNA-seq[a]

tRNA	***Ec* 34**[b]	***Ec* 37**	***Bs* 34**	***Bs* 37**	***Sa* 34**[c]	***Sa* 37**[c]	***Bv* 34**[c]	***Bv* 37**[c]
AlaGGC					x	x		
AlaTGC		m^{2}A						
ArgACG	I		I	m^{1}G	I	m^{1}G	I	m^{1}G
ArgCCG		m^{1}G		m^{1}G		m^{1}G		m^{1}G
ArgCCT			x	x	x	x		
ArgTCT								
AsnGTT		m^{2}A						
AspGTC								
CysGCA		ms^{2}i^{6}A						ms^{2}i^{6}A
fMetCAT								
GlnCTG		m^{2}A	x	x	x	x		
GlnTTG		m^{2}A			U*			
GluCTC	x	x	x	x	x	x		
GluTTC		m^{2}A						
GlyCCC			x	x	x	x		
GlyGCC								
GlyTCC								
HisGTG		m^{2}A		m^{1}G		m^{1}G		m^{1}G
IleGAT								
IleCAT	k^{2}C		k^{2}C		k^{2}C		k^{2}C	
LeuCAA	C*	ms^{2}i^{6}A		i^{6}A	C*	i^{6}A		ms^{2}i^{6}A
LeuCAG		m^{1}G		m^{1}G	x	x	C*	m^{1}G
LeuGAG		m^{1}G		m^{1}G		m^{1}G	G*	m^{1}G
LeuTAA	U*	ms^{2}i^{6}A	U*	ms^{2}i^{6}A	U*	ms^{2}i^{6}A	U*	ms^{2}i^{6}A
LeuTAG		m^{1}G		m^{1}G		m^{1}G		m^{1}G
LysCTT	x	x	x	x	x	x		ms^{2}t^{6}A
LysTTT				ms^{2}t^{6}A		ms^{2}t^{6}A		ms^{2}t^{6}A
MetCAT								
PheGAA		ms^{2}i^{6}A		ms^{2}i^{6}A		m^{1}G		ms^{2}i^{6}A
ProCGG		m^{1}G	x	x	x	x	C*	m^{1}G
ProGGG		m^{1}G	x	x	x	x		m^{1}G
ProTGG		m^{1}G		m^{1}G	U*	m^{1}G	U*	m^{1}G
SerCGA		ms^{2}i^{6}A	x	x	x	x		ms^{2}i^{6}A
SerGCT								
SerGGA								
SerTGA		ms^{2}i^{6}A		i^{6}A	U*	i^{6}A		ms^{2}i^{6}A
ThrCGT			x	x	x	x		
ThrGGT		m^{6}t^{6}A			x	x		
ThrTGT								
TrpCCA		ms^{2}i^{6}A		i^{6}A		i^{6}A		ms^{2}i^{6}A
TyrGTA		ms^{2}i^{6}A		ms^{2}i^{6}A		i^{6}A		ms^{2}i^{6}A
ValGAC					x	x	x	x
ValTAC								

a. All tRNA sequences from the Genomic tRNA database (reference 32, http://gtrnadb.ucsc.edu/). Detected modifications defined as stop+mutation fractions > 0.3. x = no annotated tRNA gene in that organism.
b. *Ec = E. coli; Bs = B. subtilis; Sa = S. aureus; Bv = B. viscericola*. I: inosine; k^{2}C: lysidine; C*,U*,G*: unknown; m^{2}A: 2-methyl-A; m^{1}G: N1-methyl-G; ms^{2}i^{6}A: 2-methylthio-N6-isopentenyladenosine; m^{6}t^{6}A: N6-methyl-N6-threonylcarbamoyladenosine; ms^{2}t^{6}A: 2-methylthio-N6-threonylcarbamoyladenosine; i^{6}A: N6-isopentenyladenosine.
c. Deduced from known *B. subtilis* and *E. coli* modifications.

Supplementary Table 2: Microbiome sequencing read counts, anticodon frequencies, seed sequences

DM-tRNA-seq

Sample	Total merged	Total tRNA	Total tRNA with anticodon	Fraction anticodon/ tRNA	# Seed sequences
LF 01	9,947,398	2,858,586	768,804	0.269	
LF 02	10,354,953	1,651,130	434,353	0.263	
LF 03	10,213,816	1,258,912	260,379	0.207	
HF 01	1,008,972	114,951	19,394	0.169	
HF 02	14,903,958	7,079,098	2,385,420	0.337	
HF 03	12,645,956	2,707,769	709,387	0.262	
LF 01_DM[a]	20,986,502	11,247,303	4,592,494	0.408	18,683
LF 02_DM	24,587,318	12,327,364	4,870,291	0.395	21,475
LF 03_DM	18,626,022	6,285,026	1,903,604	0.303	13,999
HF 01_DM	1,989,657	489,615	145,683	0.298	1,611
HF 02_DM	14,263,225	4,767,059	1,550,361	0.325	6,363
HF 03_DM	6,522,115	1,209,807	296,676	0.245	2,245

a. DM denotes for demethylase treated samples. LF = low-fat, HF = high-fat fed mouse samples.

16S-seq

Sample	Number of pairs analyzed	Merged total
LF 01	63,672	58,984
LF 02	26,585	25,959
LF 03	55,661	52,642
HF 01	62,304	58,849
HF 02	41,498	39,094
HF 03	56,363	52,682

Supplemental Table 3: Number of genomes used to generate the Gold Standard tRNA gene database at the bacterial class level

Class	Count	Class	Count
Acidimicrobiia	2	Flavobacteriia	69
Acidithiobacillia	5	Fusobacteriia	12
Acidobacteriia	6	Gammaproteobacteria	1078
Actinobacteria [a]	428	Gemmatimonadetes	2
Alphaproteobacteria	371	Gloeobacteria	2
Anaerolineae	1	Halobacteria	30
Aquificae	15	Ignavibacteria	2
Archaeoglobi	8	Methanobacteria	12
Bacilli [a]	742	Methanococci	14
Bacteroidia [a]	29	Methanomicrobia	49
Betaproteobacteria	292	Methanopyri	1
Blastocatellia	1	Mollicutes	106
Caldilineae	1	Negativicutes	8
Caldisericia	1	Nitrososphaeria	3
Chitinophagia	5	Nitrospira	6
Chlamydiia	110	Not Given	112
Chlorobia	11	Opitutae	3
Chloroflexia	6	Phycisphaerae	1
Chrysiogenetes	1	Planctomycetia	6
Chthonomonadetes	1	Rubrobacteria	2
Clostridia [a]	147	Solibacteres	1
Coriobacteriia [a]	9	Sphingobacteriia	6
Cytophagia	20	Spirochaetia	90
Deferribacteres	5	Synergistia	7
Dehalococcoidia	13	Thermococci	20
Deinococci	20	Thermodesulfobacteria	3
Deltaproteobacteria	68	Thermoleophilia	1
Dictyoglomia	2	Thermomicrobia	2
Elusimicrobia	1	Thermoplasmata	8
Endomicrobia	1	Thermoprotei	53
Epsilonproteobacteria	160	Thermotogae	25
Erysipelotrichia [a]	2	Tissierellia	5
Fibrobacteria	2	Verrucomicrobiae	1
Fimbriimonadia	1		

a. The six major classes found in the mouse gut microbiome by tRNA-seq and 16S-seq.

Supplementary Table 4: Anticodon assignment for decoding based on the simplest wobble consideration in bacteria.

Codon (anticodon)			
UUU (GAA)<br>UUC (GAA)<br>UUA (UAA)<br>UUG (CAA, UAA)	UCU (GGA)<br>UCC (GGA)<br>UCA (UGA)<br>UCG (CGA, UGA)	UAU (GUA)<br>UAC (GUA)<br>UAA (Stop)<br>UAG (Stop)	UGU (GCA)<br>UGC (GCA)<br>UGA (Stop)<br>UGG (CCA)
CUU (GAG)<br>CUC (GAG)<br>CUA (UAG)<br>CUG (CAG, UAG)	CCU (GGG)<br>CCC (GGG)<br>CCA (UGG)<br>CCG (CGG, UGG)	CAU (GUG)<br>CAC (GUG)<br>CAA (UUG)<br>CAG (CUG, UUG)	CGU (ICG[b])<br>CGC (ICG)<br>CGA (ICG, UCG)<br>CGG (CCG)
AUU (GAU)<br>AUC (GAU)<br>AUA (CAU[a], UAU[c])<br>AUG (CAT)	ACU (GGU)<br>ACC (GGU)<br>ACA (UGU)<br>ACG (CGU, UGU)	AAU (GUU)<br>AAC (GUU)<br>AAA (UUU)<br>AAG (CUU, UUU)	AGU (GCU)<br>AGC (GCU)<br>AGA (UCU)<br>AGG (CCU, UCU)
GUU (GAC)<br>GUC (GAC)<br>GUA (UAC)<br>GUG (CAC, UAC)	GCU (GGC)<br>GCC (GGC)<br>GCA (UGC)<br>GCG (CGC, UGC)	GAU (GUC)<br>GAC (GUC)<br>GAA (UUC)<br>GAG (CUC, UUC)	GGU (GCC)<br>GGC (GCC)<br>GGA (UCC)<br>GGG (CCC, UCC)

a. C34 in Ile(CAU) is C in the genome, but modified to lysidine.
b. A34 in Arg(ACG) is modified to inosine (I) which shows as G in sequencing.
c. These shown in red are more prevalent in eukaryotic tRNAs, and are less common in bacterial genomes.
